# Is there a hierarchy in mental health stigma?

**DOI:** 10.1192/bjo.2022.618

**Published:** 2022-12-12

**Authors:** Marco Mula

**Affiliations:** Institute of Medical and Biomedical Education, St George's University of London, London, UK; and Atkinson Morley Regional Neuroscience Centre, St George's University Hospitals NHS Foundation Trust, London, UK

**Keywords:** Stigma and discrimination, schizophrenia, depression, comorbidity, community mental health teams

## Abstract

People with mental illness fight not just against their condition but also against stigma, discrimination and inequalities. Research into stigma in mental illness has increased over time, but data on transdiagnostic stigma are still very limited. This commentary focuses on this topic alongside a recently published article in *BJPsych Open*.

**Figure:**
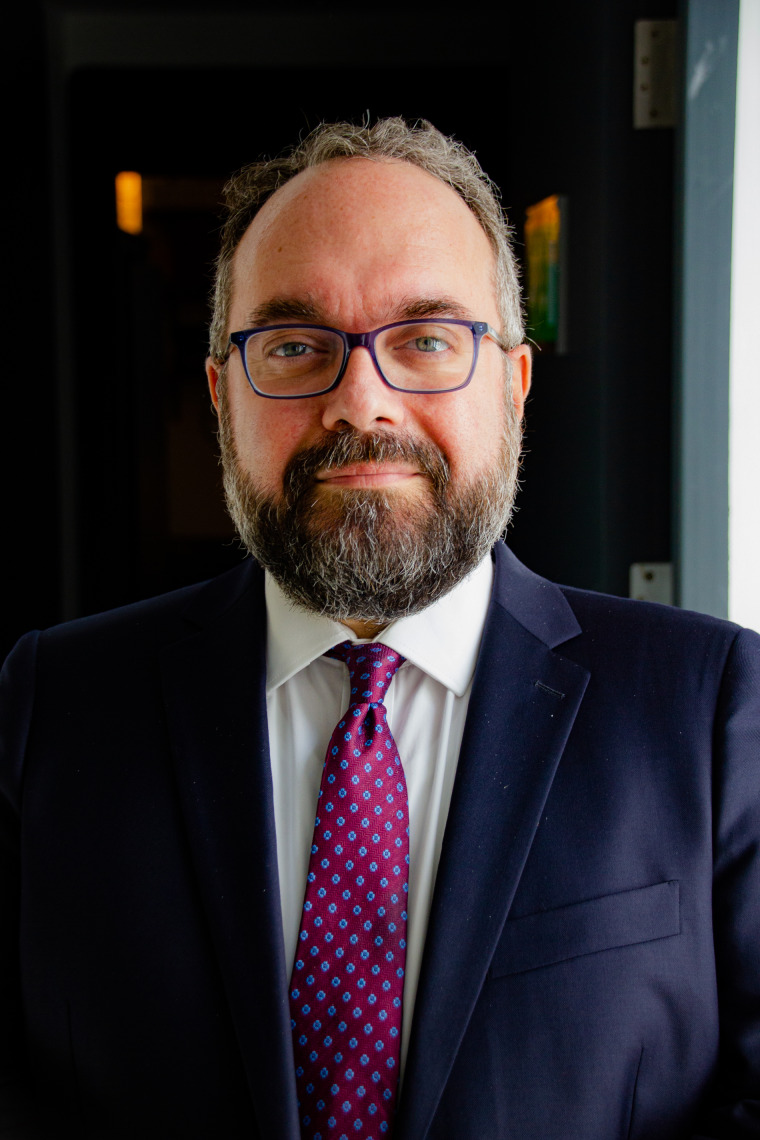

There is no doubt that there is stigma around mental illness, and this is surprising considering how common mental health problems are. Data from the Centres for Disease Control and Prevention (CDC) show that more than 50% of people in the USA are diagnosed with a mental illness at some point in their lifetime,^1^ and, according to the World Health Organization (WHO), around 350 million people in the world suffer from depression, a condition that is responsible for more ‘years lost to disability’ than any other medical condition.^2^ So why is mental illness still so stigmatised, given that it is so prevalent?

## Sources of stigma

Stigma attached to mental illness can come from several sources, such as personal, social and family beliefs, as well as a reaction to the illness itself, which may cause a person to act outside what is considered the social or cultural norm. Furthermore, self-perception of people with mental health problems or even the unconscious bias of health professionals may result in stigma. A study from the USA on healthcare providers (both primary care and mental healthcare) from the US Veterans Affair medical system looked at the relationship between mental illness stigma and healthcare decisions in relation to a vignette describing in a male patient with schizophrenia who was seeking help for low back pain due to arthritis.^3^ The study found that health practitioners who endorsed stigmatising characteristics of the patient were more likely to believe that he would not adhere to treatment and less likely to refer to a pain specialist or refill the prescription for pain medication. Sadly, the analysis showed no difference between primary care and mental health practitioners. This study clearly highlights the power of unconscious bias even among health professionals, despite research suggesting that medication and other prescription adherence of patients with psychiatric disorders is not different from what is seen in medical practice.^4^ This study also poses a few questions. One above all is about the patient's diagnosis. Would results have been the same if the patient had depression instead of schizophrenia? Are some mental health problems more stigmatised than others?

The study by Hazell et al published recently in *BJPsych Open* specifically focused on this point and investigated between-diagnosis variability in stigma.^5^

## Hazell et al's study

Hazell et al conducted an online survey in which 665 members of the public rated levels of stigma and attributions they felt towards case vignettes describing nine persons, each with a different psychiatric condition.^5^ The authors found that schizophrenia and antisocial personality disorder were associated with the highest levels of stigma, whereas depression, generalised anxiety disorder and obsessive–compulsive disorder were the least stigmatised. This study has the important role of clearly showing that stigma is not equivalent across psychiatric diagnoses and the correlates of this stigma may also be diagnosis specific. These findings clearly suggest that anti-stigma interventions and campaigns should be diagnosis specific or at least consider the complexities of individual conditions.

## Outcome data on anti-stigma interventions

There is a plethora of research on stigma in mental illness but robust outcome data are still limited. A meta-analysis of outcome studies shows that education of the public and contact with persons with mental illness have positive effects on reducing stigma,^6^ but a systematic review of evidence for the effectiveness of interventions in the longer term found only modest evidence for effectiveness beyond 4 weeks and no evidence supporting social contact as the more effective type of intervention in the medium to long term.^7^ Whether this applies to specific psychiatric diagnoses is unknown. A study looking at changes in mental illness stigma over 30 years in Germany reported that attitudes towards someone with schizophrenia became consistently more negative over that time, especially in the decade between 1990 and 2000, whereas the same did not happen for depression.^8^ There are therefore differences in stigma dynamics among different mental health problems and trajectories are also different in a changing society.

## Study limitations and future research

The study by Hazell and collaborators^5^ has many limitations, but they can act as the starting point for future research. For example, there is an obvious selection bias in their sample because most responders were employed/students, educated, White and women. Future studies will need to clarify whether the same hierarchy of stigma is present in other cultural, ethnic and religious backgrounds as well as gender groups. Studies of transcultural aspects of stigma in mental illness are still limited and it will be important to identify peculiarities in specific cultural groups in order to develop targeted anti-stigma campaigns. Future studies will need to clarify whether education and ‘mental health literacy’, as named by the authors, affect or modify this hierarchy and what mechanisms are operant. Another limitation of this study is that the authors looked at stigma in terms of social distance and attribution models and although this approach can be informative in identifying specific fear-related misbeliefs that could potentially be targeted in anti-stigma campaigns, it does not consider many other aspects such as, for example, blame, sympathy, and perceived persistence or perceived seriousness of the illness, and all these aspects can potentially contribute to the hierarchy in different ways.

Finally, stigma in mental illness should not be considered as a single entity. People with mental illnesses are complex human beings with different ages, genders, sexual orientations, ethnic and religious background, as well as disabilities and medical comorbidities, and all these factors should be considered as they may well be burdened by discrimination and stigma themselves. This phenomenon is well-known as ‘double stigma’ and it is still underappreciated. Double stigma has been identified in relation to mental illness in ethnic minorities^9^ and in people with well-known stigmatised conditions such as epilepsy,^10^ HIV or obesity, but it can be present when other discriminatory dynamics are operant, such as ageism or ableism. Systematic research in this area is urgently needed.

## Fighting stigma

The WHO Mental Health Action Plan,^11^ ratified by the World Health Assembly in May 2013, clearly states its vision for the future:

‘A world in which mental health is valued, promoted and protected, mental disorders are prevented and persons affected by these disorders are able to exercise the full range of human rights and to access high quality, culturally-appropriate health and social care in a timely way to promote recovery, in order to attain the highest possible level of health and participate fully in society and at work, free from stigmatization and discrimination.’

However, this will remain largely aspirational if required funding for mental health is not allocated. Almost half of the world's population lives in a country with only two psychiatrists per 100 000 people.^2^ Mental health professionals should be the starting point, along with patients and their families, for the development of educational initiatives, but this is not possible with such a limited and fragmented workforce. In addition, cooperation among different medical specialties and across different associations is needed to develop effective anti-stigma campaigns, because it is not possible to fight stigma effectively without fighting against discrimination and inequalities.

## Data Availability

Data availability is not applicable to this article as no new data were created or analysed in this study.
